# Effect of concomitant usage of alteplase and mechanical thrombectomy for M1 middle cerebral artery occlusion on clinical outcome: a retrospective analysis of 457 patients from two centers

**DOI:** 10.3389/fneur.2024.1286639

**Published:** 2024-02-28

**Authors:** Slaven Pikija, Monika Killer-Oberpfalzer, Johannes A. R. Pfaff, Christoph J. Griessenauer, Michael Sonnberger, Milan Vosko, Johannes S. Mutzenbach, Daniel Schwarzenhofer, Constantin Hecker

**Affiliations:** ^1^Department of Neurology, University Hospital Salzburg, Christian Doppler Klinik, Paracelsus Medical University, Salzburg, Austria; ^2^Institute of Neurointervention, Paracelsus Medical University, Salzburg, Austria; ^3^Department of Neuroradiology, University Hospital Salzburg, Christian Doppler Klinik, Paracelsus Medical University Salzburg, Salzburg, Austria; ^4^Department of Neurosurgery, University Hospital Salzburg, Christian Doppler Klinik, Paracelsus Medical University, Salzburg, Austria; ^5^Department of Neuroradiology, Neuromed Campus, Johannes Kepler University, Linz, Austria; ^6^Department of Neurology 1, Neuromed Campus, Kepler University Hospital, Johannes Kepler University, Linz, Austria; ^7^Department of Neurology 2, Med Campus III, Kepler University Hospital, Johannes Kepler University, Linz, Austria

**Keywords:** stroke, intracerebral hemorrhage, alteplase (rt-PA), mechanical thrombectomy (MT), modified Rankin scale (mRS)

## Abstract

**Introduction:**

Endovascular thrombectomy (EVT) and concomitant usage of intravenous alteplase (alteplase) in large vessel occlusion stroke may produce unwanted excess intracerebral hemorrhage (ICH). Whether this applies specifically to isolated occlusion of the M1 segment of the middle cerebral artery (MCA) is unknown.

**Methods:**

A retrospective study from two tertiary thrombectomy centers. ICH was determined according to Heidelberg Bleeding Classification (HBC). Factors associated with the occurrence of ICH in EVT alone vs. EVT with alteplase were evaluated using logistic regression analysis. Factors related to the clinical outcome as determined with a modified Rankin scale (mRS) were investigated with univariate and adjusted multivariate logistic regression analysis. The interaction between clinical variables and the usage of alteplase on the occurrence of ICH was evaluated.

**Results:**

Any ICH occurred in 156/457 (34.1%) patients Class 1a bleeding in 37 (8.1%), type 2 in 45 (9.8%) Class 1c in 22 (4.8%), Class 2 in 25 (5.5%), and Class 3 (extraparenchymal) in 27 (5.9%). ICH occurred in similar frequency between alteplase-treated patients vs. EVT alone (85/262 [32%] vs. 71/195 [36%]; OR 1.19 (95% CI 0.81–1.76). After adjustment, odds for clinical outcome were lower in ICH patients (OR 0.44 [95% CI 0.25–0.74]), *p* = 0.002). Higher ICH rate was associated with more EVT steps (p for interaction −0.005), and usage of only stent-retriever (*p* for interaction =0.005).

**Conclusion:**

Utilization of alteplase alongside EVT for MCA M1 occlusion did not result in excessive ICH occurrences or clinical deterioration.

## Introduction

Endovascular thrombectomy (EVT) is a widely used treatment for acute anterior circulation occlusion, particularly in carefully selected patients. The procedure may be performed up to 24 h after the onset of symptoms and can be effective even in patients with large areas of ischemic core (1–4). Nevertheless, when the treatment is administered in the extended time window, there is a greater likelihood of intracranial hemorrhage (ICH) occurring (4). In the early time window (up to 6 h from symptom onset to groin puncture), EVT has been shown to have a number needed to treat (NNT) of only two patients to achieve a good functional outcome (5). Among all intracranial vessels, solitary middle cerebral artery (MCA) M1 occlusion impacts patients mostly, and from all solitary vessel occlusions, it is found to be the most frequent (28%) (6).

In the appropriate time window, alteplase is recommended as the first-line treatment for acute anterior circulation occlusion and should be administered before EVT when possible (7, 8). Intracranial hemorrhage (ICH), and particularly symptomatic ICH (sICH) are significant concerns associated with the use of alteplase. In all ischemic stroke patients, irrelevant of the presence of visible occlusion or not, up to 7% of patients receiving alteplase may experience sICH, which can have a detrimental effect on clinical outcomes (9, 10). The reperfusion of the affected brain region following EVT or alteplase treatment can increase the risk of ICH. It was shown that some types of ICH, especially PH-1 (that is, hematoma within the infarcted tissue occupying <30% without substantive mass effect) are more frequent when alteplase and EVT are concomitantly used (11). However, the occurrence of ICH is a complex process that is influenced by multiple factors, including the biological effects of alteplase on the coagulation system, reperfusion injury, and the disruption of the blood–brain barrier (12).

Known predictors for ICH after concomitant alteplase and EVT administration are higher initial National Institutes of Health Stroke Score (NIHSS), higher initial systolic blood pressure, diabetes mellitus, poor collaterals, internal carotid artery occlusion, longer procedure time, and passes of retriever >3, and modified thrombolysis in cerebral infarction score ≥ 2b was associated with a decreased risk of sICH (13).

It should be noted that the data used in the analysis were pooled for all vessels in the anterior circulation, and it is unclear whether these results can be generalized to isolated MCA M1 occlusion. Further research is needed to clarify this issue (11).

The aim of this study was to explore the relationship between alteplase administration, the occurrence of ICH, and the clinical outcomes in a large cohort of patients who underwent EVT for emergent MCA M1 occlusion. Utilizing real-world data from two tertiary centers during the contemporary era of EVT, we specifically aimed to address the following research questions:

1. Does the use of alteplase correlate with an increased incidence of ICH, as classified by the Heidelberg Bleeding Classification, in cases of emergent MCA M1 occlusion?
2. Are the administration of alteplase and the presence of different types of ICH associated with clinical outcomes following mechanical thrombectomy for MCA M1 occlusion?

## Methods

### Study design

This retrospective study took place in two comprehensive stroke centers located in Austria, Europe. The study design received approval from the local ethics committees in both centers. Informed consent was not required because the study was conducted retrospectively. Both centers keep ongoing records of all patients who undergo endovascular therapy (EVT) at their respective locations.

### Selection criteria

We recruited patients who underwent EVT for emergent solitary M1 occlusion of MCA as seen on the first non-invasive angiographic imaging without any specific exclusion criteria prior to EVT. Patients without follow-up imaging within the first 36 h after EVT were excluded from the study.

### Data collection

Patient’s demographic and clinical history, National Institutes of Health Stroke Scale (NIHSS), blood glucose level at admission, time data points, and administration of thrombolytic therapy with related data points were collected in both registries. Specifically, every effort was made to collect relevant data on the recent (within 48 h) intake of oral anticoagulation. The sources of data included (1) the patient, (2) the relatives, and (3) the medical records. The medical records in both centers were cross-referenced with medication pickup data from the pharmacy along with the withdrawal date. This strategy was employed to minimize the occurrence of missing or false-positive data. Imaging data points were obtained from initial triage imaging, where a brain computed tomography (CT) scan with CT angiography was used. The Alberta Stroke Program Early CT Score (ASPECTS) was evaluated by a noninterventionist neuroradiologist for each patient, using a diffusion-weighted-imaging sequence or CT scan (14). Treatment was performed in a dedicated neuro angiography suite with up-to-date equipment under conscious sedation or general anesthesia (GA) based on local practice. The initial angiogram allowed for occlusion site and collateral status assessment using the American Society of Interventional and Therapeutic Neuroradiology/Society of Interventional Radiology scoring system, where a score < 3 indicated poor collateral status (15). The EVT revascularization technique used either a stent retriever or a direct contact aspiration technique or a combination of both techniques, based on the discretion of the local operator. Recanalization results were evaluated using the modified Thrombolysis in Cerebral Ischemia (mTICI) score, where a score ≥ 2b at the end of the procedure indicated successful recanalization (15). Time from symptom onset to initiation of IV tPA (intravenous tissue-type plasminogen activator)/to groin puncture/to recanalization was recorded. Post-interventional imaging, primarily CT scans, conducted within 36 h was used to assess the presence of intracranial hemorrhage (ICH) with Heidelberg Bleeding Classification (HBC) (16). Any imaging performed after this 36-h window was not included in the evaluation. Per HBC, ICH is divided into three classes, comprising seven types: Class 1a = hemorrhagic infarction type 1 (HI-1), that is, scattered small petechiae, no mass effect; Class 1b = hemorrhagic infarction type 2 (HI-2), that is, confluent petechiae without mass effect; Class 1c = parenchymal hematoma type 1 (PH-1), that is, hematoma within the infarcted tissue occupying <30% without substantive mass effect; and HBC Class 2 = parenchymal hematoma type 2 (PH-2), that is, ICH within and beyond infarcted brain tissue occupying 30% or more of the infarcted tissue with obvious mass effect. ICH outside the infarcted brain tissue or intracranial-extracerebral hemorrhage was divided into parenchymal hematoma remote from infarcted brain tissue (HBC Class 3a), intraventricular hemorrhage (HBC Class 3b), subarachnoid hemorrhage (HBC Class 3c), and subdural hemorrhage (HBC Class 3d). After conducting a thorough examination (which included long-term monitoring available at each center, heart ultrasound in almost all patients, and vascular ultrasound), the stroke etiology was determined using the TOAST classification system (Trial of ORG 10172 in Acute Stroke Treatment) (17). A noninterventionist vascular neurologist or a clinical research associate with certified training in modified Rankin Scale (mRS) assessment conducted a clinical outcome assessment encounter at 90 days, which involved face-to-face interviews, and telephone conversations with the patient, their relatives, or their general practitioner. Poor clinical outcome was defined as an mRS score of ≥3.

### Statistical methods

Categorical variables were presented as frequencies and percentages. Continuous variables were expressed as mean (standard deviation) or median (interquartile range [IQR]) for non-normally distributed data. The data distribution was assessed visually and through the Shapiro–Wilk test to determine if it was normally distributed. ICH presence was evaluated with Heidelberg bleeding classification and considered in total as “any ICH,” and grouped as symptomatic ICH (defined by the National Institute of Neurological Disorders and Stroke (NINDS) criteria) (18) vs. other types of ICH (Table 1). The association between baseline characteristics, such as demographics, medical history, and stroke event characteristics, with the occurrence of any intracranial hemorrhage was evaluated. Univariate analysis was performed using Fisher’s test or chi-squared test for binary and categorical data, and the Student’s t-test or Mann–Whitney test for continuous data. Variables with a value of p less than 0.10 were selected for the multivariable model. Age, anticoagulant use, prior use of alteplase, and history of diabetes mellitus were also included in the model based on prior hypotheses and biological plausibility.

**Table 1:** Intracerebral hemorrhage according to Heidelberg Bleeding Classification across treatment groups in the patients treated with endovascular thrombectomy for emergent M1 segment middle cerebral artery occlusion in two centers, *N* = 457.

Variable		Overall, *N* = 457^1^	Alteplase and EVT, *N* = 262^1^	EVT-only, *N* = 195^1^	OR (95% CI)	Value of *p*^2^
Any hemorrhage		156 (34%)	85 (32%)	71 (36%)	1.19 (0.81, 1.76)	0.4
Symptomatic hemorrhage		21 (4.6%)	14 (5.3%)	7 (3.6%)	1.52 (0.62, 4.07)	0.4
Hemorrhage class and type						
1a	HI-1	37 (8.1%)	23 (8.8%)	14 (7.2%)	1.15 (0.58, 2.37)	0.7
1b	HI-2	45 (9.8%)	30 (11%)	15 (7.7%)	1.40 (0.73, 2.78)	0.3
1c	PH-1	22 (4.8%)	6 (2.3%)	16 (8.2%)	0.26 (0.09, 0.66)	0.007
Class 1, HI total		82 (18%)	53 (20%)	29 (15%)	1.45 (0.89, 2.41)	0.14
2	PH-2	25 (5.5%)	14 (5.3%)	11 (5.6%)	0.89 (0.39, 2.07)	0.8
Class 2, PH total		47 (10%)	20 (7.6%)	27 (14%)	0.51 (0.28, 0.94)	0.033
Extra-parenchymal (Classes 3a, 3b, 3c, and 3d)		27 (5.9%)	12 (4.6%)	15 (7.7%)	0.56 (0.25, 1.24)	0.2
No hemorrhage		301 (66%)	177 (68%)	124 (64%)	1	

^1^Median (IQR) or Frequency (%). ^2^Pearson’s Chi-squared test. ICH, intracerebral hemorrhage; HI, hemorrhagic infarctions; PH, parenchymal hematoma.

To handle missing values in the predictors, a multivariable imputation technique known as the “chained equation” method was employed. Continuous variables were imputed using predictive mean-matching, binary variables were imputed using logistic regression, and categorical variables were not imputed. Since less than 10% of the data were missing, five imputed datasets were generated. All results presented are from the pooled dataset. We used variance inflation factor to detect multicollinearity in a regression model. The value >5 was considered problematic. A backward selection approach was used to develop the prediction model using the Akaike information criterion on each of the imputed datasets. Predictors that appeared in at least half of the models were selected for the final model. Statistical environment R v. 4.2.2 was used for analysis, and *p* < 0.05 was used as statistically significant (19).

## Results

A total of 636 patients were treated due to large vessel occlusion (LVO) at the study center Linz in the period 2018–2020 (both inclusive). Of them, 219 were treated for MCA M1. At the Salzburg site, we included patients in the range 2015–2020 (both inclusive), where a total of 560 patients were treated with EVT due to LVO, of them 238 for MCA M1 occlusion, therefore, 457 patients were treated for isolated MCA M1 occlusion in both centers during the study periods (Supplemental Figure 1).

### Clinical outcome

A total of 395 patients has available 90-day mRS assessment. Good clinical outcome (mRS 0–2) was observed in 192 (48.7%). One hundred sixty patients (41%) were male. The median age was 77 [interquartile range (IQR) 66–83] years.

Good outcome was associated with lower age (72 vs. 80 years of age, *p* < 0.001), absence of arterial hypertension (86/203 [42%] vs. 59/192 [31%], *p* = 0.016), absence of diabetes (93/192 [48%] vs. 132/203 [65%], *p* < 0.001), absence of atrial fibrillation (15/192 [7.8%] vs. 29/203 [14%], *p* = 0.041), absence of oral anticoagulation (OAC) intake prior to intervention (5/192 [2.6%] vs. 20/203 [9.9%], *p* = 0.003), lower glucose level at admission (118 vs. 126 mg/dL, *p* < 0.001), lower NIHSS at admission (15.0 vs. 18.0, *p* < 0.001), lower presence of symptoms at awakening (34/192 [18%], vs. 58/203 [29%]), lower procedure time (35 vs. 54 min, *p* < 0.001), higher ASPECTS (>6) (176/191 [92%] vs. 169/199 [85%], *p* = 0.026), good leptomeningeal collaterals (130/192 [68%], vs. 109/197 [55%], *p* = 0.012), higher frequency of aspiration only EVT device usage (99/179 [55%] vs. 69/178 [39%], *p* = 0.005), lower number of EVT steps (1 vs. 2, *p* < 0.001), better mTICI outcome (2b-3 vs. 0–2a) (180/92 [94%] vs. 157/203 [77%], *p* < 0.001), absence of HI-2 type of ICH (14/192 [7.3%] vs. 26/203 [13%], *p* = 0.015) and absence of any type of hemorrhage (146/192 [76%] vs. 120/203 [59%], *p* < 0.001), all comparisons for good vs. bad clinical outcome (Supplementary Table 1).

**Figure 1:**
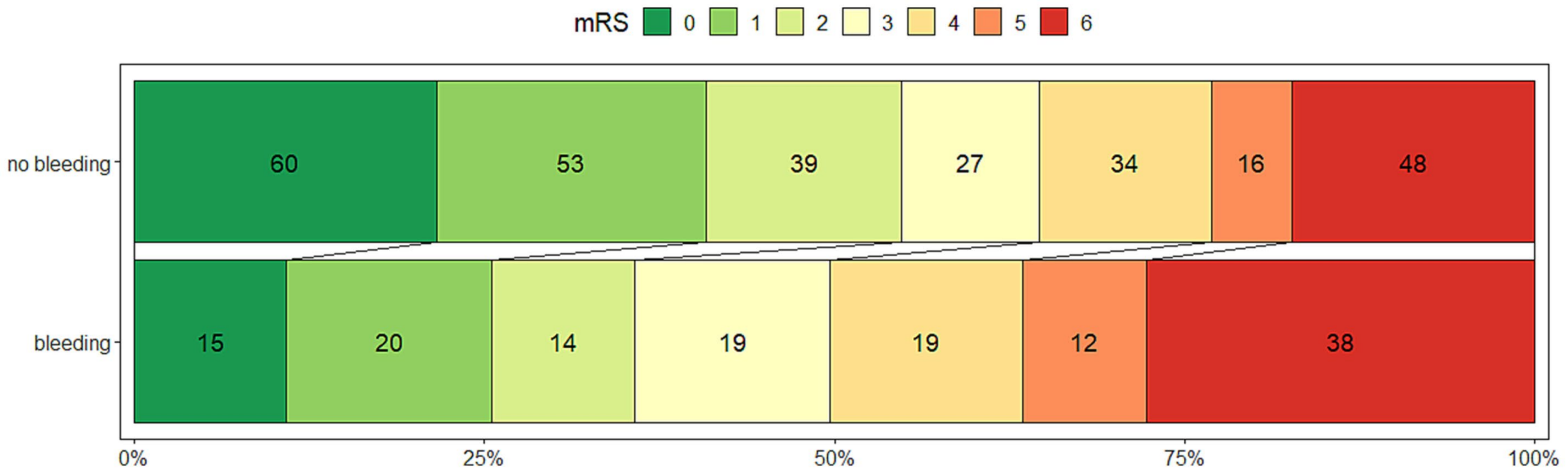
The outcome measured by the modified Rankin Scale at 90 days after thrombectomy for M1 segment middle cerebral artery occlusion, stratified by the presence of any intracranial hemorrhage after 08–36 h, in 395 patients.

When the association of variables was examined with univariate logistic regression, the patients with ICH (*N* = 266) had a lower rate of a good outcome, 46/129 (35.6%) vs. 146/266 (54.8%), OR 0.46 (0.29–0.70), *p* < 0.001 (Figure 1). Among the ICH patients, symptomatic ICH had the worst clinical outcome; only 2 (1.1%), OR 0.19 (0.03–0.71) achieved a good outcome (Table 2).

**Table 2:** Multivariate logistic regression analysis on 395 patients for good clinical outcome [assessed by modified Rankin Scale (mRS) at 3 months, good outcome: 0–2], treated with endovascular thrombectomy due to emergent M1 segment middle cerebral artery occlusion at two centers.

		mRS 0–2	
Characteristic	*N* (%)	OR 95% CI	*p*-value
No ICH (*N* = 266)	146 (35.6%)	1	NA
ICH (*N* = 129)	46 (54.8%)	0.46 (0.29–0.70)	<0.001
Symptomatic (*N* = 18)	*N* = 2 (4.3%)	0.19 (0.03–0.71)	0.032
**ICH**			
HI Total (*N* = 70)	*N* = 27 (38.5%)	1.32 (0.64–2.76)	0.5
PH Total (*N* = 35)	*N* = 11 (31.4%)	0.77 (0.33–1.74)	0.5
Extraparenchymal (*N* = 24)	*N* = 8(33.3%)	0.88 (0.33–2.20)	0.8

CI, confidence interval; ICH, intracerebral hemorrhage; HI, hemorrhagic infarctions; PH, parenchymal hematoma.

After adjustments for age, OAC, glucosis per mg/dl increase, NIHSS at admission, per point increase, presence of known time for onset of symptoms, more than the score 6 on ASPECTS, number of EVT steps performed, and EVT outcome, any ICH was significantly associated with less odds for a good outcome, aOR 0.44 (0.25–0.74), *p* = 0.002 (Table 2).

### Radiological outcome

ICH occurred in 156/457 (34%) of patients. PH-1 was significantly less present in patients with alteplase (6/262 [2.3%] vs. 16/195 [8.2%], *p* = 0.007, for those with alteplase vs. only EVT group). In univariate logistic regression analysis, odds for PH-1 were higher for patients taking OAC (OR 3.51 [0.96–10.2], *p* = 0.033), and with a higher number of EVT steps performed, (2 [IQR 1–3] vs. 1 [IQR 1–3] [no PH-1], OR 1.25 [1.00–1.52], *p* = 0.035).

### Alteplase in MCA M1 occlusion

The alteplase was administered in 262 (57%) patients. The patients treated with alteplase were significantly younger (76 vs. 79, *p* = 0.004) and male (47% vs. 69%, *p* = 0.013). Atrial fibrillation (AFib) was present or detected in 170 (37%) of patients and more common in those not treated with alteplase (89/195 [46%] vs. 81/262 [31%], *p* = 0.001). Patients with AFib were more common in older individuals (80 vs. 73 years of age, *p* < 0.001) and in women (109/170 [64%] vs. 61/170 [36%], *p* = 0.041). They also exhibited a higher frequency of OAC and direct anticoagulation (DOAC) usage (25/170 [15%] vs. 5/287 [1.7%] and 44/170 [26%] vs. 2/287 [0.7%] for OAC vs. DOAC, respectively, p < 0.001 for both).

The patients with atrial fibrillation (AFib) also experienced a less favorable outcome at 3 months, with 41% (59 out of 145) compared to 53% (133 out of 250) in the non-AFib group (*p* = 0.016). The alteplase-treated group showed a higher frequency of a history of previous ischemic stroke (18% vs. 12%, *p* = 0.048). Additionally, the intake of OAC and DOAC was lower in the alteplase group, both for patients with a history of OAC (2.7% vs. 12%) and those with a history of DOAC (4.2% vs. 18%), both *p* < 0.001.

Despite these differences, the alteplase group demonstrated a better good outcome at 3 months (64% vs. 52%, *p* = 0.023). The presence of any intracranial hemorrhage (ICH) and parenchymal hematoma (PH-2) did not differ between the groups. However, there were numerically more hemorrhagic infarctions type 2 (HI-2) in alteplase-treated patients (11% vs. 7.7%).

Several factors were identified as interacting with alteplase, resulting in higher ICH rates in patients treated with both alteplase and endovascular therapy (EVT). These factors included a history of OAC intake (aOR 10.6 [2.09–70.9] vs. aOR 1.64 [0.59–4.39], p for interaction = 0.007), the number of EVT steps (aOR 1.30 [1.09–1.56] vs. aOR 1.24 [1.02–1.51], p for interaction = 0.005), and the usage of only stent-retriever (aOR 4.22 [1.46–8.62] vs. aOR 0.53 [0.16–1.60], p for interaction = 0.005; Supplementary Tables 1, 3).

## Discussion

This two-center contemporary retrospective study shows that in the event of emergent solitary MCA M1 occlusion and after EVT treatment: (1) More than one-third of patients (34%) developed any ICH; (2) concomitant treatment with alteplase is not associated with overall ICH frequency; and (3) after adjustment for confounding factors, clinical outcome was worse in the event of ICH.

Several randomized and observational studies investigated whether alteplase with EVT conjures higher ICH risk.

The study by Hu et al. included 591 patients from the DIRECT-MT trial, and they reported intracranial hemorrhage (ICH) based on Heidelberg Bleeding Classification (HBC) (11). The MCA M1 segment was involved in about 51.7% of patients. The overall occurrence of ICH was 43.0%, with symptomatic ICH (sICH) accounting for 5.4%. In our population, the rate of ICH was smaller than in Hu et al.’s study. The frequency of hemorrhagic infarction and parenchymal hematoma was similar between the two studies, but the association with clinical outcomes differed. The use of alteplase in Hu et al.’s study showed a significant association with PH, unlike in our study where the absence of alteplase was associated with PH. Similar to our study presence of ICH and sICH was associated with less odds for a good outcome as measured by mRS (0–2); however in contrast with Hu et al. study, we did not found an association of HI or PH with clinical outcome.

The DEVT Randomized clinical trial, investigating alteplase simultaneously with EVT in 239 patients, found a similar occurrence of sICH in EVT alone vs. alteplase + EVT groups (20). The SKIP randomized trial (204 patients) compared alteplase + EVT with EVT alone, favoring EVT alone based on a lower rate of ICH (21).

Results from the MR CLEAN–NO IV trial and a randomized study by Fischer et al. favored alteplase + EVT (22, 23). Mitchell et al. reported that EVT + alteplase was non-inferior to EVT alone (24). Boisseau et al.’s multicenter registry (1,316 patients) found parenchymal hematoma to be associated with a lower rate of favorable outcomes and increased mortality (25). Zhang et al.’s observational study (629 patients) identified various predictors of sICH but did not find alteplase to be a significant predictor (26).

The referenced randomized trials showed in the majority non-inferiority of combination therapy and most of them reports no significantly higher frequency of ICH combination therapy. The differences regarding primary outcome – non-inferiority of EVT alone, between the studies are most likely explained by a combination of various factors, including ethnicity, sample size, prior use of antiplatelet or anticoagulation therapy, and the duration between onset of symptoms and intravenous alteplase administration.

Above mentioned reports have a variable frequency of MCA M1 occlusion in patients treated with EVT, ranging from 51% up to 83%. Most comparable to our results is, therefore DEVT study, where indeed sICH occurred in marginally smaller proportion to our results 6.1% vs. 3.6% for EVT alone and, 6.8% vs. 5.3% for EVT + alteplase (20). Frequency of sICH, when compared with other studies using HBC methodology, showed lower rates in comparison (DIRECT-MT 5.4%, DEVT 6.5%, and MR CLEAN–NO IV 5.6%) vs. 4.6% (11, 20, 22). The smaller number in our population is coming from the fact that we did not include ICA and M2 occlusions that could have more propensity for later hemorrhagic transformations. ICA occlusion alone or in combination with distal occlusions after EVT with and without alteplase is reported to have 8.5% sICH frequency, which is higher than any study reported above (27). M2 occlusion treated with EVT could have sICH rate of up to 10% (28, 29). Such high rates, as pointed by authors, probably arise due to technical issues during EVT in small-caliber vessels (29). This discrepancy could explain lower rate of ICH in our cohort.

Our study did not show a higher frequency of PH in the alteplase group, as in Hu et al., on the contrary, the absence of alteplase raised the frequency of PH, which can probably be explained by a higher number of patients treated with OAC and higher number of EVT steps in these cases.

According to results from our interaction analysis, in the event of a patient having a history of taking OAC, the alteplase usage is associated with the occurrence of ICH. These results could warrant caution of applying alteplase to patients having an OAC history, therefore, EVT alone should be the treatment of choice for these patients. However, our data is small in numbers and large in confidence intervals, therefore, no firm conclusion can be drawn. We could also not corroborate findings from previous interaction analysis where additional factors such are diabetes, hypertension, antiplatelet therapy, and statin administration were found to have interactions with alteplase leading to a higher frequency of ICH (Hu et al.). Again, as mentioned above, study type and different vessels probably contributed to significant discrepancies between our studies.

According to our experience, HBC is feasible to apply and could emerge as the preferred methodology in future studies, facilitating comparison between studies.

The limitations of our study are its retrospective design, however, we included patients from two centers in the contemporary period of EVT. Around 10% of patients were without information for 3-months outcome, limiting our prognostic conclusions.

## Conclusion

Based on our results, considering all limitations, we can conclude that in the event of solitary MCA M1 occlusion, alteplase is not associated with ICH and may be safe to apply.

## Data availability statement

The raw data supporting the conclusions of this article will be made available by the authors, without undue reservation.

## Ethics statement

Ethical approval was not required for the studies involving humans because of retrospective analysis of anonymized data. The studies were conducted in accordance with the local legislation and institutional requirements. Written informed consent for participation was not required from the participants or the participants’ legal guardians/next of kin in accordance with the national legislation and institutional requirements because retrospective analysis of anonymized data.

## Author contributions

SP: Conceptualization, Formal analysis, Methodology, Writing – original draft. MK-O: Conceptualization, Writing – review & editing. JP: Conceptualization, Writing – review & editing. CG: Writing – review & editing. MS: Conceptualization, Writing – review & editing. MV: Conceptualization, Writing – review & editing. JM: Conceptualization, Writing – review & editing. DS: Conceptualization, Writing – review & editing. CH: Conceptualization, Writing – review & editing.

## References

[1] Goyal M Menon BK Van Zwam WH Dippel DWJ Mitchell PJ Demchuk AM. Endovascular thrombectomy after large-vessel ischaemic stroke: a meta-analysis of individual patient data from five randomised trials. Lancet. (2016) 387:1723–31. doi: 10.1016/S0140-6736(16)00163-X, PMID: 26898852

[2] Berkhemer O Fransen P Beumer D van den Berg L Lingsma H Yoo A. A randomized trial of Intraarterial treatment for acute ischemic stroke. N Engl J Med. (2015) 372:11–20. doi: 10.1056/NEJMoa1411587 25517348

[3] Elgendy IY Kumbhani DJ Mahmoud A Bhatt DL Bavry AA. Mechanical Thrombectomy for acute ischemic stroke a Meta-analysis of randomized trials. J Am Coll Cardiol. (2015) 66:2498–505. doi: 10.1016/j.jacc.2015.09.070 26653623

[4] Yoshimura S Sakai N Yamagami H Uchida K Beppu M Toyoda K. Endovascular therapy for acute stroke with a large ischemic region. N Engl J Med. (2022) 386:1303–13. doi: 10.1056/NEJMoa2118191 35138767

[5] Martinez-Gutierrez JC Leslie-Mazwi T Chandra RV Ong KL Nogueira RG Goyal M. Number needed to treat: A primer for neurointerventionalists. Interv Neuroradiol. (2019) 25:613–8. doi: 10.1177/1591019919858733 31248312 PMC6838845

[6] Hansen CK Christensen A Ovesen C Havsteen I Christensen H. Stroke severity and incidence of acute large vessel occlusions in patients with hyper-acute cerebral ischemia: results from a prospective cohort study based on CT-angiography (CTA). Int J Stroke. (2015) 10:336–42. doi: 10.1111/ijs.12383, PMID: 25319377

[7] Berge E Whiteley W Audebert H De MGM Fonseca AC Padiglioni C. European stroke organisation (ESO) guidelines on intravenous thrombolysis for acute ischaemic stroke. Eur Stroke J. (2021) 6:I–LXII. doi: 10.1177/2396987321989865, PMID: 33817340 PMC7995316

[8] Powers WJ Rabinstein AA Ackerson T Adeoye OM Bambakidis NC Becker K. American Heart Association Stroke Council. 2018 Guidelines for the Early Management of Patients With Acute Ischemic Stroke: A Guideline for Healthcare Professionals From the American Heart Association/American Stroke Association. Stroke (2018). 49:e46–e110. doi: 10.1161/STR.0000000000000158 29367334

[9] Fiorelli M Bastianello S Von Kummer Rüdiger Del Zoppo GJ Larrue V Lesaffre E. Hemorrhagic transformation within 36 hours of a cerebral infarct relationships with early clinical deterioration and 3-month outcome in the European cooperative acute stroke study I (ECASS I) cohort. (1999). Available at: http://ahajournals.org 10.1161/01.str.30.11.2280 10548658

[10] Yaghi S Willey JZ Cucchiara B Goldstein JN Gonzales NR Khatri P. Treatment and outcome of hemorrhagic transformation after intravenous alteplase in acute ischemic stroke a scientific statement for healthcare professionals from the American Heart Association/American Stroke Association. Stroke. (2017) 48:e343–61. doi: 10.1161/STR.0000000000000152, PMID: 29097489

[11] Hu X Zhou Y Ospel J Yao F Liu Y Wang H. Intracranial hemorrhage in large vessel occlusion patients receiving endovascular thrombectomy with or without intravenous alteplase: a secondary analysis of the DIRECT-MT trial. J NeuroIntervent Surg. (2022) 10:1–7. doi: 10.1136/jnis-2022-019021 PMC10511977 36270789

[12] Jin R Yang G Li G. Molecular insights and therapeutic targets for blood-brain barrier disruption in ischemic stroke: critical role of matrix metalloproteinases and tissue-type plasminogen activator. Neurobiol Dis. (2010) 38:376–85. doi: 10.1016/j.nbd.2010.03.008, PMID: 20302940 PMC2862862

[13] Dong S Yu C Wu Q Xia H Xu J Gong K. Predictors of symptomatic intracranial hemorrhage after endovascular Thrombectomy in acute ischemic stroke: a systematic review and Meta-analysis. Cerebrovasc Dis. (2022) 52:363–75. doi: 10.1159/000527193 36423584

[14] Barber PA. Erratum: validity and reliability of a quantitative computed tomography score in predicting outcome of hyperacute stroke before thrombolytic therapy [the lancet (2000) (1670)]. Lancet. (2000) 355:2170. doi: 10.1016/s0140-6736(00)02237-6 10905241

[15] Higashida RT Furlan AJ Roberts H Tomsick T Connors B Barr J. Trial design and reporting standards for intra-arterial cerebral thrombolysis for acute ischemic stroke. Stroke. (2003) 34:e109–37. doi: 10.1161/01.STR.0000082721.62796.09, PMID: 12869717

[16] Von Kummer R Broderick JP Campbell BCV Demchuk A Goyal M Hill MD. The Heidelberg bleeding classification: classification of bleeding events after ischemic stroke and reperfusion therapy. Stroke. (2015) 46:2981–6. doi: 10.1161/STROKEAHA.115.010049 26330447

[17] Adams HP Jr Bendixen BH Kappelle LJ Biller J Love BB Gordon DL. Classification of subtype of acute ischemic stroke. Definitions for use in a multicenter clinical trial. TOAST. Trial of org 10172 in acute stroke treatment. Stroke. (1993) 24:35–41. doi: 10.1161/01.STR.24.1.35 7678184

[18] National Institute of Neurological Disorders and Stroke rt-PA Stroke Study Group. Tissue plasminogen activator for acute ischemic stroke. N Engl J Med. (1995) 333:1581. doi: 10.1056/NEJM199512143332401 7477192

[19] Team RC. R: A language and environment for statistical computing. Vienna, Austria: R Foundation for Statistical Computing (2021).

[20] Zi W Qiu Z Li F Sang H Wu D Luo W. Effect of endovascular treatment alone vs intravenous Alteplase plus endovascular treatment on functional Independence in patients with acute ischemic stroke: the DEVT randomized clinical trial. JAMA. (2021) 325:234–43. doi: 10.1001/jama.2020.23523, PMID: 33464335 PMC7816099

[21] Suzuki K Matsumaru Y Takeuchi M Morimoto M Kanazawa R Takayama Y. Effect of mechanical Thrombectomy without vs with intravenous thrombolysis on functional outcome among patients with acute ischemic stroke: the SKIP randomized clinical trial. JAMA. (2021) 325:244–53. doi: 10.1001/jama.2020.23522, PMID: 33464334 PMC7816103

[22] LeCouffe NE Kappelhof M Treurniet KM Rinkel LA Bruggeman AE Berkhemer OA. A randomized trial of intravenous Alteplase before endovascular treatment for stroke. N Engl J Med. (2021) 385:1833–44. doi: 10.1056/NEJMoa2107727 34758251

[23] Fischer U Kaesmacher J Strbian D Eker O Cognard C Plattner PS. Thrombectomy alone versus intravenous alteplase plus thrombectomy in patients with stroke: an open-label, blinded-outcome, randomised non-inferiority trial. Lancet. (2022) 400:104–15. doi: 10.1016/S0140-6736(22)00537-2, PMID: 35810756

[24] Mitchell PJ Yan B Churilov L Dowling RJ Bush SJ Bivard A. Endovascular thrombectomy versus standard bridging thrombolytic with endovascular thrombectomy within 4·5 h of stroke onset: an open-label, blinded-endpoint, randomised non-inferiority trial. Lancet. (2022) 400:116–25. doi: 10.1016/S0140-6736(22)00564-5 35810757

[25] Boisseau W Fahed R Lapergue B Desilles JP Zuber K Khoury N. Predictors of parenchymal hematoma after mechanical Thrombectomy: a multicenter study. Stroke. (2019) 50:2364–70. doi: 10.1161/STROKEAHA.118.024512, PMID: 31670928

[26] Zhang X Xie Y Wang H Yang D Jiang T Yuan K. Symptomatic intracranial hemorrhage after mechanical Thrombectomy in Chinese ischemic stroke patients: the ASIAN score. Stroke. (2020) 51:2690–6. doi: 10.1161/STROKEAHA.120.030173 32811387

[27] Díaz-Pérez J Parrilla G Espinosa De Rueda M Cabrera-Maqueda JM García-Villalba B Alba-Isasi MT. Mechanical Thrombectomy in acute stroke due to carotid occlusion: a series of 153 consecutive patients. Cerebrovasc Dis. (2018) 46:130–9. doi: 10.1159/000492866, PMID: 30212823

[28] Saber H Narayanan S Palla M Saver JL Nogueira RG Yoo AJ. Mechanical thrombectomy for acute ischemic stroke with occlusion of the M2 segment of the middle cerebral artery: a meta-analysis. J Neurointerv Surg. (2018) 10:620–4. doi: 10.1136/neurintsurg-2017-013515, PMID: 29127196

[29] de Castro Afonso LH Borghini Pazuello G Seizem Nakiri G Monsignore LM Antunes Dias F Pontes-Neto OM. Thrombectomy for M2 occlusions and the role of the dominant branch. Interv Neuroradiol. (2019) 25:697–704. doi: 10.1177/1591019919847693, PMID: 31088246 PMC6838850

