# Supplementary material for: Effect of concomitant usage of alteplase and mechanical thrombectomy for M1 middle cerebral artery occlusion on clinical outcome: a retrospective analysis of 457 patients from two centers
Source: Front Neurol. 2024 Feb 28;15:1286639. doi: 10.3389/fneur.2024.1286639 (PMC10933087; doi:10.3389/fneur.2024.1286639)
Supplement: Supplementary file 1 [file Data_Sheet_1.pdf]

		Modified Rankin Scale at 3 months		
**Variable**	**Overall**, N = 395[1]	**0-2**, N = 192[1]	**3-6**, N = 203[1]	**p-value**[2]
**Age (years)**	77 (66, 83)	72 (60, 79)	80 (73, 85)	<0.001
**Gender, man**	160 (41%)	85 (44%)	75 (37%)	0.14
**Study site**				0.33
Linz	165 (42%)	85 (44%)	80 (39%)	
Salzburg	230 (58%)	107 (56%)	123 (61%)	
**Arterial Hypertension**	145 (37%)	59 (31%)	86 (42%)	0.016
**Diabetes**	225 (57%)	93 (48%)	132 (65%)	<0.001
**Atrial fibrillation**	44 (11%)	15 (7.8%)	29 (14%)	0.041
**History of statin use**	66 (17%)	32 (17%)	34 (17%)	0.98
**Ischemic heart disease**	64 (16%)	31 (16%)	33 (16%)	0.98
**History of ischemic stroke**	59 (15%)	23 (12%)	36 (18%)	0.11
**Intake of antiplatelet therapy prior to event**	77 (19%)	34 (18%)	43 (21%)	0.38
**Intake of OAC therapy prior to event**	25 (6.3%)	5 (2.6%)	20 (9.9%)	0.003
**Intake of DOAC therapy prior to event**	37 (9.4%)	17 (8.9%)	20 (9.9%)	0.73
**Glucosis (mg/dl)***	121 (107, 145)	118 (104, 135)	126 (109, 160)	<0.001
**NIHSS at admission**[#]	17.0 (12.0, 20.0)	15.0 (10.0, 19.0)	18.0 (15.0, 20.0)	<0.001
**Symptoms at awakening**	92 (23%)	34 (18%)	58 (29%)	0.011
**ASPECTS > 6**[†]	345 (88%)	176 (92%)	169 (85%)	0.026
**Good or equal Leptomeningeal collaterals**[β]	239 (61%)	130 (68%)	109 (55%)	0.012
**TOAST**[¥]				0.022
CE	253 (64%)	109 (57%)	144 (71%)	
LAA	46 (12%)	28 (15%)	18 (8.9%)	

Other	8 (2.0%)	3 (1.6%)	5 (2.5%)	
Undetermined	3 (0.8%)	2 (1.0%)	1 (0.5%)	
Unknown	84 (21%)	50 (26%)	34 (17%)	
**Alteplase**	228 (58%)	122 (64%)	106 (52%)	0.023
**Type of anestesia[£]**				0.21
CS	33 (8.4%)	19 (9.9%)	14 (6.9%)	
CS than GS	2 (0.5%)	2 (1.0%)	0 (0%)	
GA	358 (91%)	171 (89%)	187 (93%)	
Local	1 (0.3%)	0 (0%)	1 (0.5%)	
**Symptom onset to groin puncture (min)**	190 (145, 250)	192 (143, 258)	190 (149, 242)	0.49
Unknown	104	40	64	
**Procedure time (min)**	42 (24, 71)	35 (20, 56)	54 (30, 78)	<0.001
**Type of EVT device**				0.005
aspiration + stent retriever	145 (41%)	59 (33%)	86 (48%)	
aspiration only	168 (47%)	99 (55%)	69 (39%)	
Stent retriever only	44 (12%)	21 (12%)	23 (13%)	
Unknown	38	13	25	
**First passage with EVT device successfull[µ]**	166 (44%)	105 (55%)	61 (32%)	<0.001
Unknown	14	2	12	
**Total thrombectomy steps performed**	2.00 (1.00, 3.00)	1.00 (1.00, 3.00)	2.00 (1.00, 3.00)	<0.001
Unknown	10	2	8	
**mTICI outcome**				<0.001
2b-3	337 (85%)	180 (94%)	157 (77%)	
0-2a	58 (15%)	12 (6.2%)	46 (23%)	
**Ischemic lesion volume**	14 (2, 61)	4 (1, 21)	39 (6, 117)	<0.001
Unknown	6	2	4	
**Symptomatic hemorrhage**	18 (4.6%)	2 (1.0%)	16 (7.9%)	0.001

**Heidelberg bleeding classification**				0.015
HI-1	30 (7.6%)	13 (6.8%)	17 (8.4%)	
HI-2	40 (10%)	14 (7.3%)	26 (13%)	
PH-1	17 (4.3%)	6 (3.1%)	11 (5.4%)	
PH-2	18 (4.6%)	5 (2.6%)	13 (6.4%)	
Extraparenchymal hemorrhage	24 (6.1%)	8 (4.2%)	16 (7.9%)	
**Presence of any type of hemorrhage**				<0.001
hemorrhage	129 (33%)	46 (24%)	83 (41%)	
no hemorrhage	266 (67%)	146 (76%)	120 (59%)	

NIHSS – National Institutes of Health Scale; OAC – oral Vitamin-K anticoagulation therapy; DOAC – direct oral anticoagulation therapy; ASPECTS - Alberta Stroke Program Early CT Score; TOAST - Trial of ORG 10172 in Acute Stroke Treatment; CS – conscious sedation; GA – general anesthesia; Local – local anesthesia at groin puncture site; EVT – endovascular thrombectomy; mTICI – modifed Thrombolysis in Cerebral Infarction scale; mRS – modified Rankin Scale; ICH – intracerebral hemorrhage

* missing in 6.1% of patients; # missing in 5.3%; † missing in 1.3%; β missing in 1.8%; ¥ missing in 0.7%; £ missing in 0.7%; § missing in 1.5%; µ missing in 3.5%; α missing in 2.4%; ε missing in 1.5%; ¶ missing in 13.6%.

Supplemental Table 1. Demographic characteristics of 395 patients treated with endovascular thrombectomy for emerging M1 occlusion of medical cerebral artery.

	Univariate regression model			Multivariate regression model		
Characteristic	OR[1]	95% CI[1]	p-value	aOR	95% CI	p-value
Age (years)	0.95 (0.93, 0.97)		<0.001	0.94	0.92, 0.96	<0.001
Arterial Hypertension	0.51	0.34, 0.76	<0.001			
Atrial fibrillation	0.60	0.40, 0.91	0.017			
Intake of OAC therapy prior to event	0.24	0.08, 0.62	0.006	0.24	0.07, 0.73	0.016
Glucosis (mg/dl)	0.99	0.98, 0.99	<0.001	0.99	0.98, 0.99	<0.001
NIHSS at admission	0.90	0.86, 0.93	<0.001	0.90	0.86, 0.94	<0.001
Symptoms at awakening	0.54	0.33, 0.86	0.011	0.51	0.27, 0.96	0.037
ASPECTS > 6[†]	2.08	1.10, 4.11	0.028	2.21	0.94, 5.34	0.073
Good or equal Leptomeningeal collaterals[β]	1.69	1.12, 2.56	0.012			
Alteplase	1.59	1.07, 2.39	0.023			
Type of EVT device						
aspiration + stent retriever	—	—				
aspiration only	2.09	1.33, 3.30	0.001			
Stent retriever only	1.33	0.67, 2.63	0.4			
Procedure time (min)	1.00	1.0, 1.00	0.3			
Total thrombectomy steps performed	0.82	0.72, 0.93	0.002	0.78	0.66, 0.92	0.004
mTICI outcome 0-2a	0.23	0.11, 0.43	<0.001	0.36	0.15, 0.82	0.019
Symptomatic ICH	0.12	0.02, 0.44	0.006			
Any ICH	0.46	0.29, 0.70	<0.001	0.44	0.25, 0.74	0.002

Supplemental Table 2. Univariate and multivariate logistic regression on clinical outcome after 3 months in 395 patients treated with endovascular thrombectomy at two centers for emergent M1 middle cerebral artery occlusion.

		alteplase given		
Variable	Overall, N = 457[1]	yes, N = 262[1]	no, N = 195[1]	p-value[2]
**Age (years)**	77 (65, 83)	76 (63, 82)	79 (68, 85)	0.004
**Gender, man**	192 (42%)	123 (47%)	69 (35%)	0.013
**Study site**				0.50
Linz	219 (48%)	122 (47%)	97 (50%)	
Salzburg	238 (52%)	140 (53%)	98 (50%)	
**Arterial Hypertension**	262 (57%)	153 (58%)	109 (56%)	0.59
**Diabetes**	54 (12%)	25 (9.5%)	29 (15%)	0.081
**Atrial fibrillation**	170 (37%)	81 (31%)	89 (46%)	0.001
**History of statin use**	77 (17%)	43 (16%)	34 (17%)	0.77
**Ischemic heart disease**	77 (17%)	42 (16%)	35 (18%)	0.59
**History of ischemic stroke**	67 (15%)	31 (12%)	36 (18%)	0.048
**Intake of antiplatelet therapy prior to event**	86 (19%)	58 (22%)	28 (14%)	0.035
**Intake of OAC therapy prior to event**	30 (6.6%)	7 (2.7%)	23 (12%)	<0.001
**Intake of DOAC therapy prior to event**	46 (10%)	11 (4.2%)	35 (18%)	<0.001
**Glucosis***	122 (108, 148)	121 (106, 147)	122 (109, 150)	0.40
**NIHSS at admission[#]**	17.0 (12.0, 20.0)	17.0 (12.0, 20.0)	16.0 (12.0, 19.0)	0.96
**Symptoms at awakening**	109 (24%)	45 (17%)	64 (33%)	<0.001
**ASPECTS > 6[†]**	396 (88%)	238 (93%)	158 (81%)	<0.001
**Good or equal Leptomeningeal collaterals[β]**	274 (61%)	158 (61%)	116 (61%)	0.91
**TOAST[¥]**				0.015
CE	292 (64%)	152 (58%)	140 (73%)	
LAA	50 (11%)	33 (13%)	17 (8.8%)	
Other	9 (2.0%)	5 (1.9%)	4 (2.1%)	

Undetermine d	3 (0.7%)	3 (1.1%)	0 (0%)	
Unknown	100 (22%)	68 (26%)	32 (17%)	
**Type of anestesia£**				0.77
CS	35 (7.7%)	19 (7.3%)	16 (8.2%)	
CS than GS	2 (0.4%)	1 (0.4%)	1 (0.5%)	
GA	418 (92%)	242 (92%)	176 (91%)	
Local	1 (0.2%)	0 (0%)	1 (0.5%)	
**Symptom onset to groin puncture (min)**	197 (150, 259)	199 (150, 250)	194 (148, 289)	0.54
Unknown	123 (26.9%)	54 (20.6%)	69 (35.3%)	
**Procedure time (min)§**	40 (24, 70)	40 (24, 66)	43 (23, 74)	0.58
**Type of EVT device**				>0.99
aspiration + stent retriever	170 (41%)	95 (41%)	75 (41%)	
aspirarion only	187 (45%)	105 (46%)	82 (45%)	
Stent retriever only	54 (13%)	30 (13%)	24 (13%)	
Unknown	46 (10.0%)	32 (12.2%)	14 (7.1%)	
**First passage with EVT device successfullµ**	195 (44%)	113 (45%)	82 (43%)	0.63
Unknown	16	12	4	
**Total thrombectomy steps performedα**	1.00 (1.00, 3.00)	1.00 (1.00, 3.00)	2.00 (1.00, 3.00)	0.22
Unknown	11	9	2	
**mTICI outcome**				0.053
2b-3	393 (86%)	232 (89%)	161 (83%)	
0-2a	63 (14%)	29 (11%)	34 (17%)	
**mRS outcome after 3 months¶**				0.023
0-2	192 (49%)	122 (54%)	70 (42%)	
3-6	203 (51%)	106 (46%)	97 (58%)	

Unknown	62	34	28	
**Any ICH**				0.38
hemorrhage	156 (34%)	85 (32%)	71 (36%)	
no hemorrhage	301 (66%)	177 (68%)	124 (64%)	

NIHSS – National Institutes of Health Scale; OAC – oral Vitamin-K anticoagulation therapy; DOAC – direct oral anticoagulation therapy; ASPECTS - Alberta Stroke Program Early CT Score; TOAST - Trial of ORG 10172 in Acute Stroke Treatment; CS – conscious sedation; GA – general anesthesia; Local – local anesthesia at groin puncture site; EVT – endovascular thrombectomy; mTICI – modified Thrombolysis in Cerebral Infarction scale; mRS – modified Rankin Scale; ICH – intracerebral hemorrhage

* missing in 6.1% of patients; # missing in 5.3%; † missing in 1.3%; β missing in 1.8%; ¥ missing in 0.7%; £ missing in 0.7%; § missing in 1.5%; μ missing in 3.5%; α missing in 2.4%; ε missing in 1.5%; ¶ missing in 13.6%.

Supplemental Table 3. Demographic characteristics of 457 patients treated with endovascular thrombectomy for emerging M1 occlusion of medical cerebral artery, grouped by usage of intravenous thrombolysis.

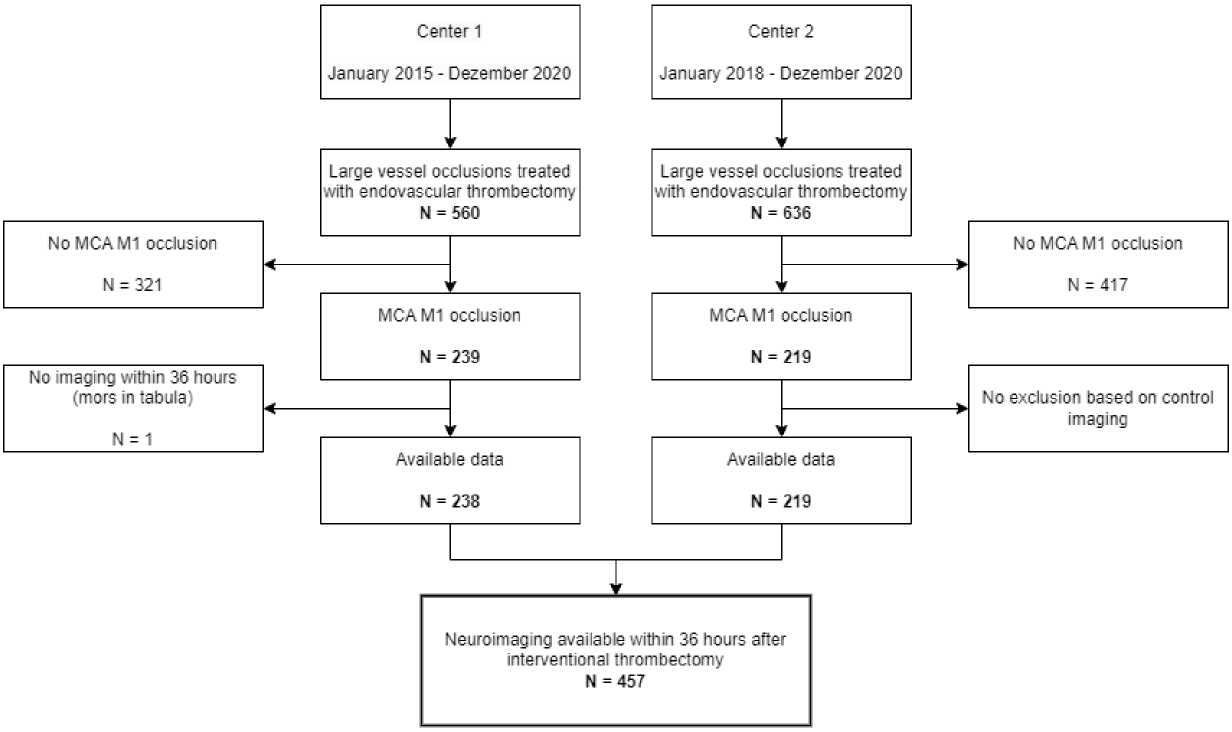

Supplemental Figure 1. Patient flow diagram in two centers 2015 (2018) – 2022. There were one exclusion event (1/239 [0.41%]) in the Salzburg group due to death during the intervention.
